# Clinical value of station 4R node dissection in esophageal squamous cell carcinoma

**DOI:** 10.1186/s12957-023-03280-7

**Published:** 2023-12-18

**Authors:** Xin-ye Wang, Xia-yu Fu, Hong Yang, Jing Wen, Peng Lin, Jian-hua Fu

**Affiliations:** 1 Department of Thoracic Surgery, Sun Yat-Sen University Cancer Center, 651 Dongfeng Road East, Guangzhou, 510060 China; 2 Guangdong Esophageal Cancer Institute, Collaborative Innovation Centre of Cancer Medicine, State Key Laboratory of Oncology in South China, Guangzhou, 510060 China

**Keywords:** Esophageal squamous cell carcinoma (ESCC), Station 4R, Clinical value

## Abstract

**Background:**

Many controversies still exist concerning the optimal extent of lymphadenectomy during esophagectomy in esophageal squamous cell carcinoma (ESCC). The objective of this study was to explore the characteristics of 4R metastasis and evaluate the clinical value of 4R node dissection in ESCC.

**Methods:**

A total of 736 ESCC patients who underwent radical esophagectomy between 2005 and 2013 were retrospectively collected, among which 393 ones underwent 4R dissection. Propensity score matching (PSM) method was applied to reduce the effects of confounding variables between the 4R dissection and non-dissection groups to analyze overall survival.

**Results:**

Patients showed a low 4R metastasis rate of 5.1% (20/393) (5.2%, 5.8%, and 1.8% for upper, middle, and lower tumors, respectively). Correlation analyses identified that 4R metastasis was significantly associated with station 2R metastasis (*p* < 0.001) and pathologic tumor-node-metastasis (pTNM) stage (*p* < 0.001). All 4R metastases were observed in stages IIIB and IVA. Moreover, patients with station 4R dissection failed to achieve significantly improved overall survival compared with those without 4R dissection, regardless of tumor stage (overall: *p* = 0.696; stage 0-IIIA:* p* = 0.317; stage IIIB-IVA:* p* = 0.619).

**Conclusion:**

4R metastasis is likely to be associated with more aggressive disease, and routine 4R node dissection might not be necessary for ESCC patients.

## Introduction

Esophageal cancer is one of the most prevalent malignant tumors in China, with 477.9 million new cases. It was the fourth leading cause of cancer death in China in 2015 [1]. Esophageal squamous cell carcinoma (ESCC) is the prevailing histologic subtype in China, comprising over 90% of esophageal carcinoma cases. In contrast, the predominant type in Western countries is adenocarcinoma [2]. Surgical resection is still the cornerstone of treatment for both early-stage ESCC and locally advanced ESCC despite recent progress in multidisciplinary approaches; radical lymph node dissection is a critical part of esophagectomy with respect to optimal staging and may have a favorable impact on disease control and long-term survival [3–7]. However, extensive lymph node dissection may result in surgical trauma and increase the incidence of postoperative complications [8, 9]. Some researchers had suggested recently that lymph node dissection for some specific stations-subcarinal lymph nodes [10], common hepatic nodes [11] and splenic nodes [12]-could be skipped in certain patients. Due to the lack of unified standards, the optimal extent of lymphadenectomy is still controversial.

Lymph node metastasis is frequently observed in the upper mediastinal region, even in cases when the tumor is located in the middle or lower thoracic esophagus. Previous studies have emphasized the importance of lymphadenectomy in this area for achieving a better oncological effect [13–15]. The upper mediastinal lymph nodes consist of multiple lymph node stations, with bilateral recurrent laryngeal nerve (RLN) lymph nodes being the subject of most earlier research. Bilateral RLN lymph nodes have been reported to harbor the highest incidence of involvement (18.0%—46.5%), and the dissection of which has been verified to significantly increase the survival rate [16, 17]. In the mediastinum, station 4R lymph nodes (right lower paratracheal nodes, between the intersection of the caudal margin of the brachiocephalic artery with the trachea and the apex of the lung) are surrounded by complicated structures, such as the trachea, bronchus, pulmonary arteries, superior vena cava, and azygos [18]. In contrast, the metastasis of station 4R lymph nodes was rare in clinical practice and they presented an obviously lower metastasis rate (approximately 5%) than the bilateral RLN lymph nodes, according to a few studies [17, 19]. Therefore, we assume that the 4R lymph nodes may have distinct characteristics of metastasis from bilateral RLNs, even though these nodes are all defined as upper mediastinal lymph nodes. However, little research has been done to date to clarify the precise role of 4R lymph nodes, and it is yet unknown if routine 4R dissection improves the prognosis of thoracic ESCC patients.

In this retrospective study, we explored the characteristics of 4R metastasis and compared the long-term outcomes between the 4R dissection group and non-dissection group, with the aim to clarify the clinical value of 4R dissection in thoracic ESCC patients.

## Methods

### Study cohort

**Fig. 1:**
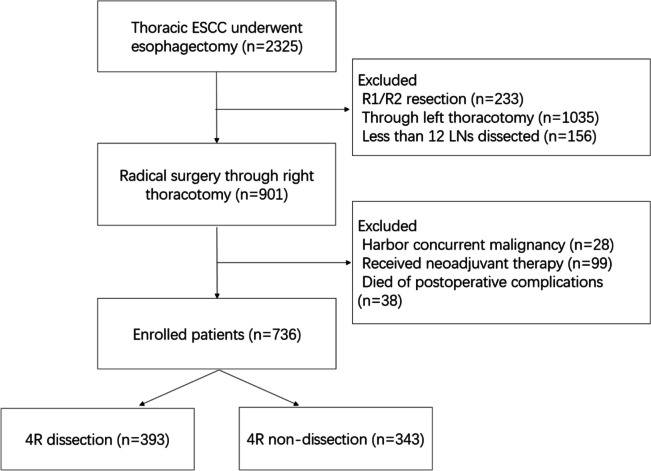
Flowchart of patients’ selection in this study. ESCC, esophageal squamous cell carcinoma; LN, lymph nodes

This study was approved by the Ethics Committee of the Sun Yat-sen University Cancer center, which waived the requirement for written informed consent from individual patients owing to the retrospective nature of this study. The data of ESCC patients who underwent radical esophagectomy between 2005 and 2013 in Sun Yat-sen University Cancer center were retrospectively collected. Then all ESCC patients were divided into a 4R dissection group (dissection group) and a non-4R dissection group depending on whether the patients underwent 4R lymph node dissection. Patients were eligible for this study if they met the following criteria: (a) aged ≥ 18 years old and with a Karnofsky performance score ≥ 90; (b) with a pathological confirmation of ESCC and a tumor located in the thoracic esophagus; (c) receiving a surgical procedure performed through right thoracotomy (Ivor-Lewis, McKeown or Akiyama), with at least 12 lymph nodes removed during lymphadenectomy; (d) undergoing radical resection (R0 resection); (e) without distant metastasis; and (f) no reception of neoadjuvant therapy. Patients were excluded if they met the following criteria: (a) with other concurrent malignant diseases; and (b) who died within 30 days after surgery or died of postoperative complications. Finally, 736 patients who met criteria were enrolled for analysis (Fig. 1).

Preoperative routine staging examinations included enhanced computed tomography (CT) of neck, chest and upper abdomen, gastroduodenoscopy or endoscopic ultrasound (EUS), and barium esophagography. Positron emission tomography/computed tomography (PET/CT) was performed if there were signs of distant metastasis. The tumor-node-metastasis (TNM) stage was assessed according to the American Joint Committee on Cancer (AJCC) cancer staging manual (8th edition) [20].

## Surgical procedure and classification of lymph node stations

Eligible patients were treated with esophagectomy with total two-field lymphadenectomy (2-FL) through right thoracotomy (Ivor-Lewis or McKeown procedure) or three-field lymphadenectomy (3-FL, Akiyama procedure). 3-FL was selectively performed when there was an indication of cervical lymph node metastasis according to the preoperative assessment. Minimally invasive esophagectomy or open surgery was chosen depending on the surgeon's preference. At least 12 lymph nodes were removed during lymphadenectomy for accurate staging [21]. The extent of 2-FL involved total mediastinal nodes (bilateral upper/lower paratracheal nodes, subcarinal nodes, upper/middle/lower thoracic para-esophageal lymph nodes, diaphragmatic nodes) and upper abdominal nodes (paracardial nodes, left gastric nodes), while 3-FL included additional dissection of bilateral lower cervical nodes on the basis of 2-FL. All patients received bilateral RLN exploration during operation. After surgery, all resected lymph nodes were labeled according to their anatomical sites and were sent for pathologic examination.

The upper mediastinal lymph nodes consist of multiple lymph node stations [stations 2R (right upper paratracheal nodes), 4R (right lower paratracheal nodes), 2L (left upper paratracheal nodes), 4L (left lower paratracheal nodes) and 8U (upper para-esophageal nodes)] based on the AJCC cancer staging manual (8th edition) [19]. In this study, we combined the stations 2L and 4L to one group for analysis, as they are usually resected and sent for pathologic examination as one block clinically. The stations 2L and 4L lymph nodes have been considered equivalent to No.106-recL (left RLN lymph nodes) in the Japanese Classification of Esophageal Cancer (JCEC) staging system (11th edition)[22–24]. Moreover, station 2R is equivalent to No.106-recR (right RLN lymph nodes), whereas station 4R is equivalent to No.106-pre (pre-tracheal lymph nodes) + No.106-tbR (right tracheobronchial lymph nodes) in the JCEC classification [23, 24].

## Follow-up

A postoperative follow-up assessment was carried out every 3 months for the first year after surgery, every 6 months for the second to fifth years, and once a year thereafter. The routine examinations included a physical examination, tumor biomarker tests, computed tomography scan, gastroduodenoscopy, and barium esophagography. Positron emission tomography/computed tomography (PET/CT) and bone scans were performed if there were signs of metastasis. In this study, 12/2018 was the last follow-up date for the survival analysis. The median follow-up period for the entire cohort was 62.5 months (range 1.31–164.8 months).

## Propensity score matching (PSM)

PSM was performed to reduce the effects of confounding variables and balance the distribution of baseline covariates between the 4R dissection group and non-dissection group [25]. The propensity scores were calculated with a multivariable logistic regression model, with the following covariates: sex, age, tumor location, surgical approach, histological grade, tumor length, pT stage, pN stage, number of dissected nodes, and postoperative treatment. After a 1:1 matching with the ‘nearest neighbor’ algorithm (caliper value: 0.05), 226 patients in each group were successfully matched.

## Statistical analysis

All statistical analyses were conducted by using SPSS 23.0 software (IBM Corp. in Armonk, NY). Chi-square tests or Fisher’s exact tests were used for categorical variables, and t-tests were used for continuous variables. Overall survival (OS) was defined as the time interval between the date of surgery and the date of either death from any reason or the last follow-up. OS was estimated by the Kaplan–Meier method and compared by the log-rank test. A multivariate Cox proportional hazards regression model was used to identify independent prognostic factors. In all analyses, a two-tailed *p* < 0.05 was considered statistically significant.

## Results

### Baseline data of patients before matching

A total of 736 patients were finally enrolled, including 393 with 4R dissection (dissection group) and 343 without 4R dissection (non-dissection group). Before matching, significant baseline differences were observed between the two groups in terms of age (*p* = 0.001), tumor location (*p* = 0.018), surgical approach (*p* < 0.001), pN stage (*p* = 0.015), pTNM stage (*p* = 0.013) and number of dissected nodes (*p* < 0.001) (Table 1).

**Table 1:** Clinicopathologic characteristics of the 4R node dissection and non-dissection groups

Variable	Before matching			After matching		
	**Dissection (*n* = 393)**	**Non-dissection (*n* = 343)**	***p***	**Dissection (*n* = 226)**	**Non-dissection (*n* = 226)**	***p***
Sex			0.654			0.823
Male	311 (79.1)	266 (77.6)		176 (77.9)	173 (76.5)	
Female	82 (20.9)	77 (22.4)		50 (22.1)	53 (23.5)	
Age (years)			**0.001**			0.819
≤ 65	323 (82.2)	247 (72.0)		176 (77.9)	179 (79.2)	
> 65	70 (17.8)	96 (28.0)		50 (22.1)	47 (20.8)	
Tumor location			**0.018**			0.849
Upper	58 (14.8)	58 (16.9)		39 (17.3)	44 (19.5)	
Middle	278 (70.7)	211 (61.5)		152 (67.3)	148 (65.5)	
Lower	57 (14.5)	74 (21.6)		35 (15.5)	34 (15.0)	
Histologic grade			0.352			0.868
G1	63 (16.0)	61 (17.8)		39 (17.3)	43 (19.0)	
G2	218 (55.5)	172 (50.1)		116 (51.3)	116 (51.3)	
G3	112 (28.5)	110 (32.1)		71 (31.4)	67 (29.6)	
Surgical approach			** < 0.001**			0.683
Mckeown + 2FL	259 (65.9)	281 (81.9)		180 (79.6)	187 (82.7)	
Akiyama + 3FL	94 (23.9)	14 (5.0)		20 (8.8)	16 (7.1)	
Ivor-lewis	40 (10.2)	45 (13.1)		26 (11.5)	23 (10.2)	
pT stage			0.098			0.783
T1a-T1b	42 (10.7)	43 (12.5)		30 (13.3)	25 (11.1)	
T2	66 (16.8)	75 (21.9)		44 (19.5)	42 (18.6)	
T3	274 (69.7)	210 (61.2)		145 (64.2)	149 (65.9)	
T4	11 (2.8)	15 (4.4)		7 (3.1)	10 (4.4)	
pN stage			**0.015**			0.843
N0	188 (47.8)	148 (43.1)		109 (48.2)	108 (47.8)	
N1	106 (27.0)	110 (32.1)		66 (29.2)	72 (31.9)	
N2	66 (16.8)	72 (21.0)		41 (18.1)	35 (15.5)	
N3	33 (8.4)	13 (3.8)		10 (4.4)	11 (4.9)	
pTNM stage			**0.013**			0.971
0	1 (0.3)	4 (1.2)		1 (0.4)	2 (0.9)	
Ia	1 (0.3)	3 (0.9)		1 (0.4)	1 (0.4)	
Ib	36 (9.2)	35 (10.2)		26 (11.5)	20 (8.8)	
IIa	53 (13.5)	45 (13.1)		33 (14.6)	36 (15.9)	
IIb	103 (26.2)	66 (19.2)		51 (23.5)	53 (22.6)	
IIIa	18 (4.6)	28 (8.2)		11 (4.9)	14 (6.2)	
IIIb	146 (37.2)	146 (42.6)		91 (40.3)	87 (38.5)	
IVa	35 (8.9)	16 (4.7)		12 (5.3)	13 (5.8)	
Tumor length (mm)			0.337			0.510
≤ 35	195 (49.6)	183 (53.4)		116 (51.3)	108 (47.8)	
> 35	198 (50.4)	160 (46.6)		110 (48.7)	118 (52.2)	
No. of dissected nodes	40.2 ± 16.7	26.6 ± 11.6	** < 0.001**	31.5 ± 11.6	30.1 ± 12.2	0.200
Postoperative treatment			0.430			0.976
None	305 (77.6)	271 (79.0)		181 (80.1)	179 (79.2)	
Radiotherapy	1 (0.3)	4 (1.2)		1 (0.4)	1 (0.4)	
Chemotherapy	84 (21.4)	66 (19.2)		43 (19.0)	45 (19.9)	
Chemo-radiotherapy	3 (0.8)	2 (0.6)		1 (0.4)	1 (0.4)	

*2FL* two-field lymphadenectomy, *3FL* three-field lymphadenectomy, *pTNM* pathologic tumor-node-metastasis. Data presented as number of patients (%) or mean ± standard deviation (SD)

## Prevalence of 4R lymph node metastasis

**Fig. 2:**
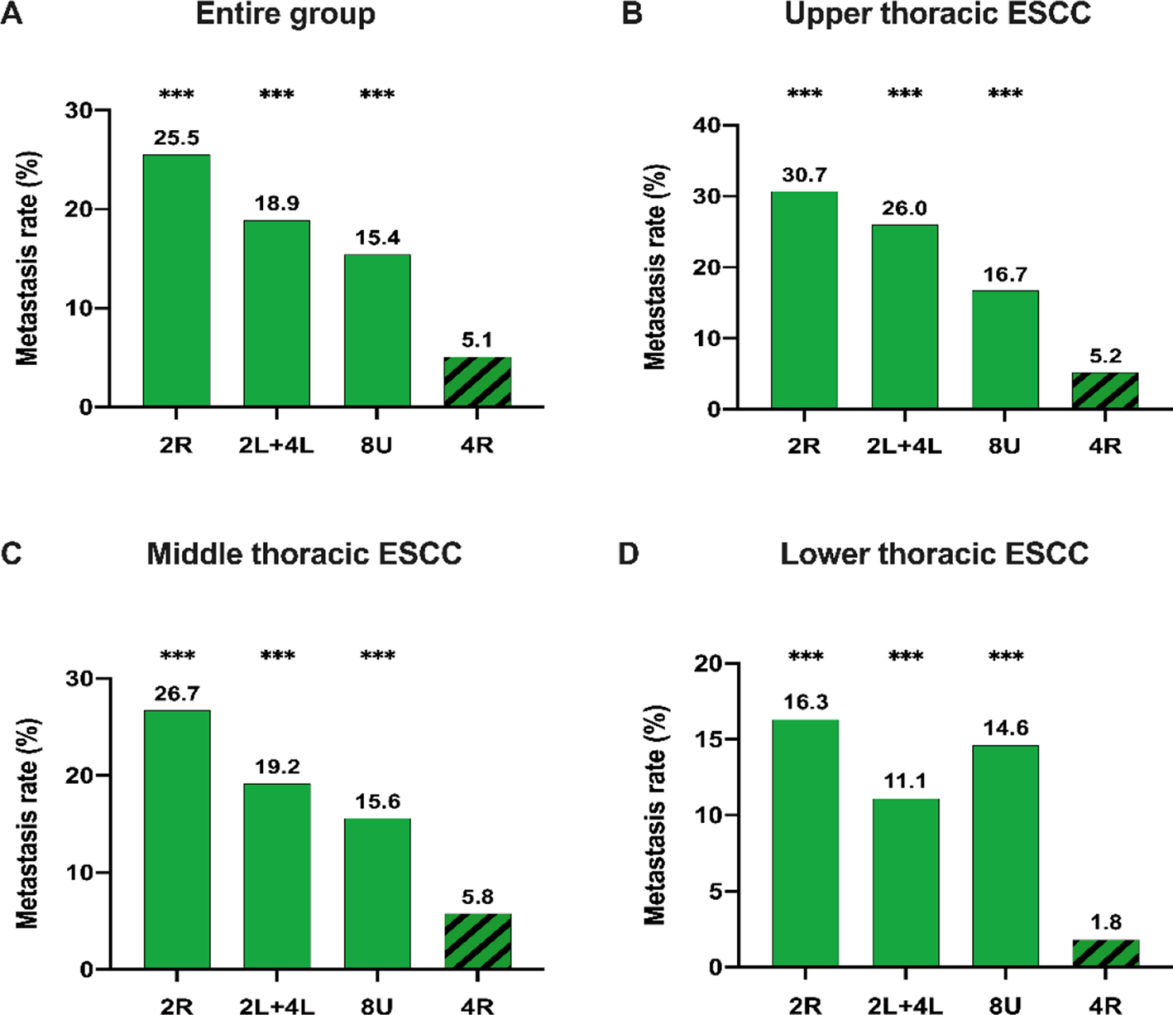
Metastasis rates of lymph node stations in the upper mediastinal region: in the entire group **(A)**; upper thoracic ESCC **(B)**; middle thoracic ESCC **(C);** lower thoracic ESCC **(D)**. The chi-square test was used to compare the metastasis rate of 4R with that of other lymph node stations. *** *p* < 0.001

Of the 393 patients in the 4R dissection group, 20 patients presented 4R metastasis; the overall metastasis rate was 5.1% (20/393), and the metastasis rate was 5.2% (3/58), 5.8% (16/278), and 1.8% (1/57) for upper, middle, and lower tumors, respectively. The 4R overall metastasis rate was much lower than the metastasis rate of other stations in the upper mediastinal region: station 2R (25.5%, *p* < 0.001), station 2L + 4L (18.9%, *p* < 0.001), and station 8U (15.4%, *p* < 0.001) (Fig. 2A). The significantly lower 4R metastasis rate was also observed when the tumors were located in the upper, middle and lower esophagus (Fig. 2B**/C/D**).

The correlation analysis of the 393 patients of the 4R dissection group showed that 4R metastasis was significantly correlated with station 2R metastasis (*p* < 0.001), pN stage (*p* < 0.001) and pTNM stage (*p* < 0.001). Sex, age, tumor location, grade, pT stage, tumor length and metastasis of other upper mediastinal nodes (2L + 4L, 8U) were not significantly correlated with 4R metastasis. Notably, no 4R metastasis occurred in patients with superficial tumors (pT1a-T1b) or high historical grades (G1); all metastases were observed in patients in stages IIIB and IVA (Table 2).

**Table 2:** Analysis of the associations between clinicopathological factors and 4R node metastasis (*n* = 393)

**Variable**	**n**	**4R-positive** **n (%)**	**4R-negative** **n (%)**	*p*
Sex				0.922
Male	311	16 (5.1)	295 (94.9)	
Female	82	4 (4.9)	78 (95.1)	
Age (years)				0.736
≤ 65	323	17 (5.3)	306 (94.7)	
> 65	70	3 (4.3)	67 (95.7)	
Tumor location				0.408
Upper	58	3 (5.2)	55 (94.8)	
Middle	278	16 (5.8)	262 (94.2)	
Lower	57	1 (1.8)	56 (98.2)	
Histological grade				0.057
G1	63	0 (0.0)	63 (100)	
G2	218	12 (5.5)	206 (94.5)	
G3	112	8 (7.1)	104 (92.9)	
pT stage				0.317
T1a-T1b	42	0 (0.0)	42 (100)	
T2	66	3 (4.5)	63 (95.5)	
T3	274	16 (5.8)	258 (94.2)	
T4	11	1 (9.1)	10 (90.9)	
pN stage				** < 0.001**
N0	188	0 (0.0)	188 (100)	
N1	106	4 (3.8)	102 (96.2)	
N2	66	6 (9.1)	60 (90.9)	
N3	33	10 (30.3)	23 (69.7)	
pTNM stage				** < 0.001**
0	1	0 (0.0)	1 (100.0)	
Ia	1	0 (0.0)	1 (100.0)	
Ib	36	0 (0.0)	36 (100.0)	
IIa	53	0 (0.0)	53 (100.0)	
IIb	103	0 (0.0)	103 (100.0)	
IIIa	18	0 (0.0)	18 (100.0)	
IIIb	146	10 (6.8)	136 (93.2)	
IVa	35	10 (28.6)	25 (71.4)	
Tumor length (mm)				0.653
≤ 35	195	11 (5.6)	184 (94.4)	
> 35	198	9 (4.5)	189 (95.5)	
2R metastasis	85	11 (12.9)	74 (87.1)	** < 0.001**
2L + 4L metastasis	39	4 (10.3)	35 (89.7)	0.099
8U metastasis	30	3 (10.0)	27 (90.0)	0.376

## Survival analysis after matching

**Fig. 3:**
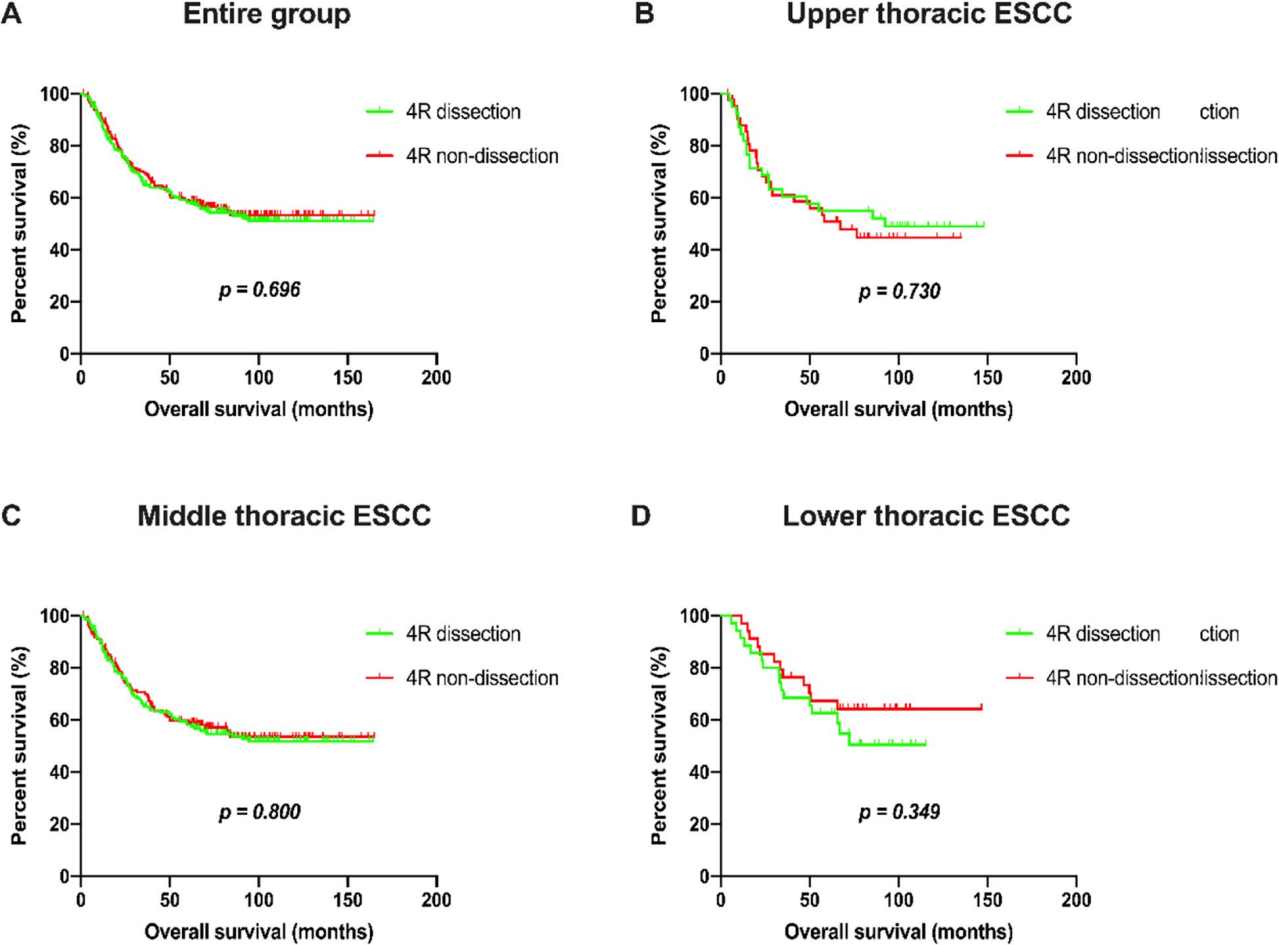
Kaplan–Meier estimates of overall survival for 4R dissection and non-dissection groups in the entire group (**A**), upper thoracic ESCC (**B**), middle thoracic ESCC (**C**) and lower thoracic ESCC (**D**)

We compared the long-term outcomes of the dissection group and non-dissection group with the PSM method to balance the baseline differences. After matching, the 226 patients in each group had comparable baseline information (*p* > 0.05) (Table 1). At the end of the follow-up period, 105 (46.5%) patients had died in the dissection group, and 97 (42.9%) had died in the non-dissection group; the OS rates in the dissection group and non-dissection group were 64.8% *vs*. 69.3% at 3 years and 58.3% *vs*. 58.7% at 5 years. There were no significant differences in OS between the two groups (*p* = 0.696) (Fig. 3A). Additionally, when stratified by tumor location, the dissection group was not superior to the non-dissection group in terms of OS (upper: *p* = 0.730; middle: *p* = 0.800; lower: *p* = 0.349) (Fig. 3B**/**C/D).

**Fig. 4:**
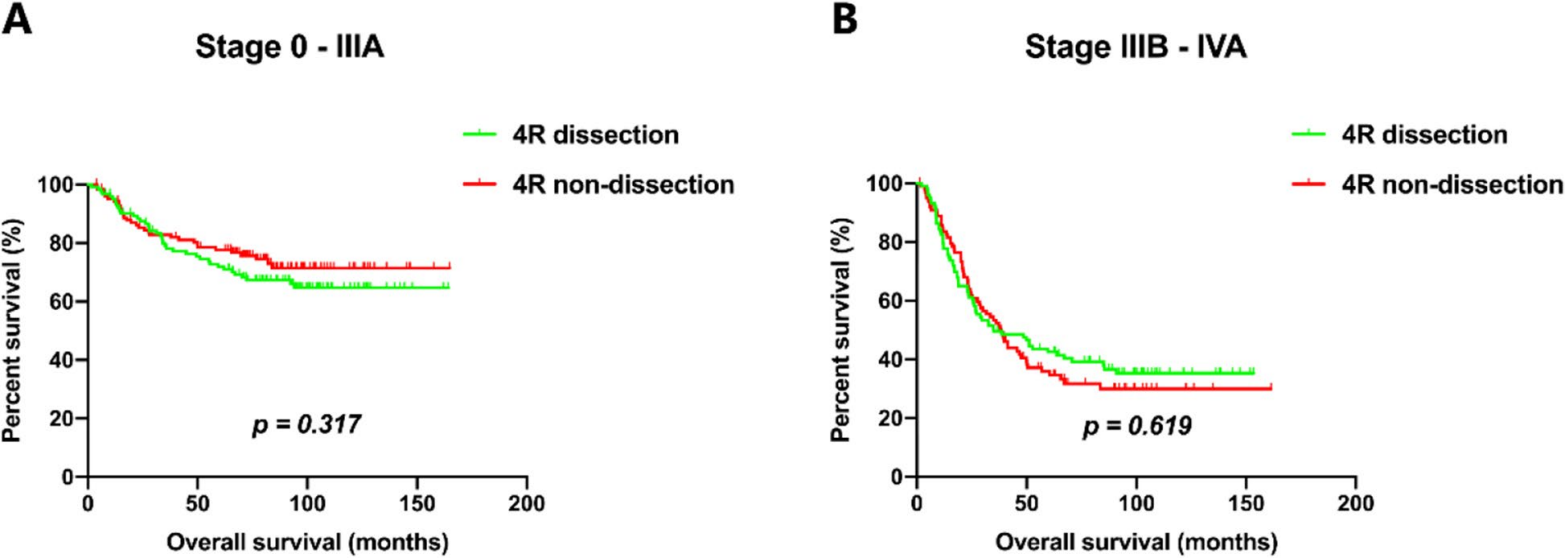
Kaplan–Meier estimates of overall survival for 4R dissection and non-dissection groups in stage 0-IIIA (**A**), and stage IIIB-IVA (**B**)

Since all 4R metastases were observed in stages IIIB and IVA, we further conducted the stratified analysis within different staging groups. As a result, regardless of patients being in stage 0-IIIA or stage IIIB-IVA, the dissection group did not significantly improve OS compared with the non-dissection group (stage 0-IIIA: *p* = 0.317; stage IIIB-IVA:* p* = 0.619) (Fig. 4).

Eventually, the multivariate Cox regression model identified that age [hazard ratio (HR) = 1.011, 95% confidence interval (CI): 1.002–1.019, *p* = 0.018], pT stage (HR = 1.641, 95% CI: 1.287–2.092, *p* < 0.001) and pN stage (HR = 1.673, 95% CI: 1.442–1.942, *p* < 0.001) were independent prognostic factors for OS (Table 3).

**Table 3:** Univariate and multivariate Cox regression analyses of overall survival in patients after matching

Variable	Univariable			Multivariable		
	**HR**	**95% CI**	***p***	**HR**	**95% CI**	***p***
Sex	0.86	0.61–1.20	0.369			
Age †	1.39	1.02–1.91	**0.040**	1.01	1.00–1.02	0.018*
Tumor location	0.83	0.66–1.05	0.126			
Histologic grade	1.10	0.90–1.34	0.364			
Surgical approach	1.04	0.85–1.29	0.686			
Tumor length	1.39	1.05–1.83	**0.021**	0.96	0.72–1.29	0.791
pT stage	1.90	1.52–2.39	** < 0.001**	1.64	1.29–2.09	** < 0.001**
pN stage	1.77	1.54–2.04	** < 0.001**	1.67	1.44–1.94	** < 0.001**
Postoperative treatment	0.98	0.88–1.10	0.753			
4R dissection status	1.06	0.80–1.39	0.696			

^†^ Time-dependent covariates

^*^statistical significance

## Discussion

Lymph node metastasis is one of the most decisive factors affecting the outcomes of ESCC patients [26]. Subtotal esophagectomy with radical lymph node dissection remains an effective therapeutic strategy for localized ESCC [6]. However, the optimal extent of lymph node dissection is still up for debate [27]. Our investigation on the value of station 4R lymph mode dissection in thoracic ESCC patients led us to several notable findings. Firstly, station 4R presented the lowest metastasis rate within the upper mediastinal region. Secondly, no 4R metastasis occurred in patients with superficial tumors (pT1a-T1b) or high historical grades (G1); all metastases were observed in stages IIIB and IVA, suggesting that 4R metastasis was likely to be associated with more aggressive disease. Moreover, survival analysis indicated that patients with station 4R dissection did not obtain significantly improved long-term survival compared with those without 4R dissection, regardless of the tumor location and the tumor stage.

Our data showed that 5.1% of the patients who underwent 4R dissection presented 4R involvement, and that the metastasis rate was only 1.8% for lower tumors; the results were in line with those of previous studies [17, 19]. The station 4R had a much lower probability of metastasis than the other upper mediastinal nodes stations, regardless of tumor location. According to our correlation analysis, 4R metastasis was significantly correlated with 2R metastasis. Furthermore, we noticed that no 4R metastases occurred in patients with superficial tumors (pT1a-T1b) and high historical grades (G1), and all of the 4R metastases were observed in patients with stage IIIB or later stage disease. Anatomically speaking, the correlations above could be explained as follows: since 2R is consecutive to the superior part of the right paratracheal lymphatic chain (PLC), there are consistent connections of extramural lymphatic drainage between 2 and 4R [28], and the lymph flow could be bidirectional [29]. Moreover, unlike 2R, which can both directly receive lymphatic drainage from abundant submucosal longitudinal vessels and from ascending extramural drainage pathways with nodal relays (including the lower stations of the right PLC) [28], 4R often relays extramural lymphatic drainage that was most likely originated at the intermuscular plexus and occasionally relays extramural lymphatic drainage from the lower nodal stations [22]. Therefore, 4R may have a small chance of being the first involved nodes. In a word, 4R metastasis is typically not observed in the early phase of the disease and may indicate a more advanced stage. These findings may suggest the necessity to perform the preoperative assessment for station 4R nodes, and 4R status should be taken into account prior to making the treatment decision.

Technically, it is not difficult to perform 4R node dissection. The para-esophageal and peri-gastric lymph nodes can be removed together with the gross specimen during esophagectomy and anatomical stomach dissociation. However, the 4R nodes need to be dissected during an extra operation, since this procedure is not directly related to tumor resection and digestive tract reconstruction and might lead to an increase in operation time, risk of blood loss and surrounding trauma. In order to improve survival, it would be ideal to remove lymph nodes that are likely to be metastasized while leaving the non-metastatic ones in situ. Omitting the dissection of a specific station on the basis of ensuring oncological outcomes could be implemented as part of the rapidly developed enhanced recovery after surgery (ERAS) protocol. In this study, we found no significant differences between the dissection group and non-dissection group regarding OS after PSM. Similar results were seen in subgroup analyses stratified by tumor location, indicating that 4R dissection might have few contributions to a better prognosis. Another remarkable finding was that the 4R dissection group did not significantly improve OS compared with the non-dissection group when stratified by stage (stage 0-IIIA: p = 0.317; stage IIIB-IVA: p = 0.619). For patients in stage IIIA-IVB, the 4R dissection alone is insufficient for the OS improvement, as have been proven by clinical trials[30] that multiple disciplinary team (MDT) comprehensive therapy, rather than surgery alone, is the suggested treatment protocol. As for those in stage 0-IIIA, the 4R dissection may be unnecessary due to the little probability of 4R metastasis.

Furthermore, there were no preoperatively available clinical factors showing a significant correlation with 4R metastasis in our study, while the historical grade seemed to be a predictive factor for metastasis risk. This may be put down to the fact that all incidences of 4R metastasis occurred in patients with G2 or G3 condition, and none in the patients with G1 condition. However, the small number of patients with 4R metastases likely contributed to the lack of significant difference (p = 0.057). Thus a larger cohort is required to further validate the correlation between historical grade and metastasis risk, and more effective preoperative markers are clinically needed.

As far as we know, this is the first study to systematically investigate the characteristics of 4R metastasis and assess the clinical value of 4R node dissection in patients with thoracic ESCC after curative esophagectomy. However, there are several limitations. Firstly, selection bias is inevitable because this is a single-center retrospective study and the decision to perform 4R node dissection depended on the surgeon’s preferences. Secondly, all of the enrolled patients underwent right thoracotomy in a single institute with strict inclusion criteria, so the data are highly homogenous. Thirdly, PSM used to balance variables might influence the prognosis of patients. In addition, the number of patients who underwent 4R dissection and had metastasis was relatively small. Hence, large prospective random controlled trials with multiple centers are needed to verify our findings. Last but not least, we excluded the patients who received neoadjuvant therapy to avoid the changes in the lymphatic drainage pattern affected by neoadjuvant treatment, so more research is needed to determine whether our results are applicable to patients who received neoadjuvant treatment.

## Conclusion

In conclusion, 4R lymph nodes present low metastasis rate in thoracic ESCC patients. 4R metastasis is likely to be associated with more aggressive disease. Routine 4R lymph node dissection might not be beneficial for ESCC patients.

## Data Availability

Datasets used in this article are available from the corresponding author on reasonable request.
